# Institutional Needs

**Published:** 1913-08-16

**Authors:** 


### Institutional Needs.

HYDROQUININE HYDROCHLORIDE.

(Vereinigte Chininfabriken Zimmer und Co., Frankfurt. London Agents: Widenmann, Broicher and Co., 1 Fenchurch Avenue, E.C.)

A quinine substitute of synthetic preparation has been placed on the market by the above firm of manufacturers. It differs from quinine by the addition of two more atoms of hydrogen per molecule, and occurs in the form of white crystals of very bitter taste, very easily soluble in water and also in alcohol, but insoluble in ether. The toxicity of this alkaloid is about the same as that of quinine, if anything slightly less. As an antimalarial specific it is said to be superior to quinine hydrochloride, and possesses as well the great advantage of much higher solubility. A future is also anticipated for hydroquinine as a trypanocidal drug, both in animals and in mankind. If these anticipations are realised, the future of hydroquinine is of course assured. Intravenous and intramuscular administration is much more conveniently carried out than with quinine salts, owing to the solubility and consequent small bulk of a given dose. Lenzmann has, it appears, been treating whooping-cough with this new drug, injected intramuscularly, with excellent results. Special ampoules containing sterile solutions of hydroquinine are now placed on the market for this purpose. Children up to six months receive 0.3 to 0.75 grain for a dose, at twelve months $1\frac{1}{2}$ grain, at two years 2 grains, and so on up to $7\frac{1}{2}$ grains at ten to fourteen years of age.

